# Food Authentication Goes Green: Method Optimization for Origin Discrimination of Apples Using Apple Juice and ICP-MS

**DOI:** 10.3390/foods13233783

**Published:** 2024-11-25

**Authors:** Marie-Sophie Müller, Marie Oest, Sandra Scheffler, Anna Lena Horns, Nele Paasch, René Bachmann, Markus Fischer

**Affiliations:** 1 Hamburg School of Food Science, Institute of Food Chemistry, University of Hamburg, Grindelallee 117, 20146 Hamburg, Germany; mariesophie.mueller@uni-hamburg.de (M.-S.M.); marie.oest@uni-hamburg.de (M.O.); sandra.scheffler@live.de (S.S.); 2 Landeslabor Schleswig-Holstein, Max-Eyth-Straße 5, 22437 Neumünster, Germany; anna-lena.horns@lsh.landsh.de (A.L.H.); nele.paasch@lsh.landsh.de (N.P.); rene.bachmann@lsh.landsh.de (R.B.)

**Keywords:** apples, apple juice, element profiling, food authentication, inductively coupled plasma mass spectrometry, chemometrics

## Abstract

Apples are among the most important fruits worldwide and the most consumed fruit in Germany. Due to higher energy and personnel costs, domestic apples are more expensive and thus offer an incentive for mixing with foreign goods. Moreover, imported apples have a higher carbon footprint, which is an obstacle regarding sales in times of climate change. Not only the transport of the goods but also the analysis influences the carbon footprint. Inductively coupled plasma mass spectrometry (ICP-MS) is a powerful tool for origin discrimination. In this study, 85 apple juice samples were analyzed, whereby sample preparation for ICP-MS was optimized by eliminating the freeze-drying step and thereby reducing CO_2_ emissions. The CO_2_ emission was lowered by around 97%. The optimized method was applied to 272 apple juice samples from seven countries to create models for origin determination. The differentiation of European and non-European apples provided an accuracy of 90.9% ± 2.4%. German samples can be differentiated from other countries with an accuracy of 83.2% ± 1.4%. The regional differentiation of German samples (north vs. south) achieved an accuracy of 92.3% ± 5.4%. The results show that the optimized ICP-MS method, in which freeze-drying is not required is well suited for determining the origin of apples from apple juice.

## 1. Introduction

The origin and, in particular, the regionality of food have become increasingly important in recent years. For example, 84% of German consumers attach importance to the regional origin when buying fruit and vegetables. Likewise, the information on the packaging regarding the origin and production conditions plays an essential role in the purchase decision [1]. Proof of origin is mainly based on delivery documents and is, therefore, susceptible to falsification. This also applies to apples, which, with a per capita consumption of 22.4 kg in 2021/22, are by far the most popular fruit in Germany [2] with an annual harvest volume of approximately one million tons [3]. In 2021, an additional 600 kt of apples was imported to Germany, which corresponds to 40% of the total volume. This makes Germany the second-largest apple importer worldwide [4]. The most important apple-producing countries from which Germany imports its apples are France, Italy, Chile, South Africa and New Zealand [5]. About 24% of the annual harvest is used as processing and industrial fruit, for example, for the production of fruit juice or canned food [6]. At 5.1 L per capita, apple juice is the second most popular fruit juice in Germany. In connection with the spritzer category, it is the most consumed juice (8.8 L per capita) [7]. The best-selling apple varieties on the German market in 2023 were Elstar with a share of 19.8% and Red Prince with a share of 12.1% [8].

In addition to price differences, which provide an incentive for food fraud, apples from different origins differ mainly in terms of their carbon footprint. The carbon footprint of an apple imported from New Zealand is almost twice as high as that of an apple grown regionally, e.g., in Schleswig-Holstein (Germany). This also takes into account the CO_2_ emissions caused by the storage and cooling of the apples [9]. Due to the high per capita consumption and the high share of imported fruit, an analytical verification of the regional origin of apples is of great importance for an improved monitoring. Furthermore, energy consumption and CO_2_ emissions should also be taken into account when developing an analytical method. Reducing CO_2_ emissions will lower greenhouse gas emissions and thus mitigate the effects on climate change. Without the use of methods with reduced carbon dioxide emission, average temperatures will rise faster, leading to more extreme weather conditions, which in turn will result in high crop losses [10].

The geographical origin of apples has already been investigated in various studies. Wenck et al. [11] studied the geographical origin of 217 apple juice samples from different countries using ^1^H NMR spectroscopy. Bat et al. [12] analyzed primary and secondary metabolites of 64 apple juice samples from Slovenia. Fifty-eight apple juice samples from different regions of China were analyzed in a study by Guo et al. [13] based on the polyphenolic profiles. However, the metabolome is susceptible to environmental influences, but also for endogenous factors, while the element profile is less sensitive.

Inductively coupled plasma mass spectrometry (ICP-MS) is particularly well suited for geographical origin determination, as plants obtain their nutrients from the soil in which they grow. Thus, they have a characteristic elemental or isotopic profile depending on the soil composite, and the generated elemental profiles can be used to create a reference-based method for determining geographical authenticity [14,15,16,17,18]. In contrast to metabolomic approaches where an influence through the variety can be recognized [11], the influence of the variety regarding element profiling approaches is negligible [15]. Sample preparation for ICP-MS analyses usually involve freeze-drying of the samples prior to acid digestion to determine accurate element concentrations that are not affected by varying water contents. This allows the comparison of total element concentrations between samples [19,20,21]. Several studies analyzing apples and apple juices were performed using ICP-MS for origin discrimination. Zhang et al. [22] studied the elemental and metabolic fingerprint of 235 apple samples from two regions in China. Apples from northwestern Italy were analyzed to evaluate the distribution and bioaccumulation of specific elements using ICP-MS [23]. Bat et al. [24] analyzed 64 apple juice samples from Slovenia using ICP-MS in combination with IR-MS. Liu et al. [25] also used the combination of ICP-MS and IR-MS to determine the origin of 384 apple juice samples from China. But, to our knowledge, no study investigated the origin determination of apple juice samples on a worldwide and regional scale using ICP-MS so far.

In this study, an ICP-MS method was developed to distinguish the origin of apples based on apple juice from different regions. The high-water content of the samples (about 85%) is associated with an extremely long drying time and thus an enormous energy consumption for freeze-drying. Therefore, an alternative analytical method was developed to avoid freeze-drying. This involves using a drying scale to determine the water content to calculate quantitative element concentrations. A metabolomic approach has already investigated the impact of freeze-drying for raw milk [26]. Moreover, different evaluation approaches were applied disregarding the water content altogether and thus reducing the experimental effort. Chemometric methods were applied to the data to create classification models for various origin discrimination problems. Additionally, the use of apple juice was verified, analyzing the corresponding apple marc of a subset of apple juices.

## 2. Materials and Methods

### 2.1. Materials

Hydrogen peroxide (H_2_O_2_, suprapur^®^, 30%) and nitric acid (HNO_3_, ROTIPURAN^®^ Supra, 69%) were purchased from Merck KGaA (Darmstadt, Germany) and Carl Roth GmbH & Co. KG (Karlsruhe, Germany). Ultrapure water (>18 MΏ) was received from a Direct Q purifying system (Merck Millipore Inc., Billerica, MA, USA). For external calibration, various multi-elemental standard solutions were purchased from Merck KGaA (Darmstadt, Germany), Carl Roth GmbH & Co. KG (Karlsruhe, Germany) and PerkinElmer Inc. (Waltham, MA, USA). Single elemental solutions (Ge, Rh, In, Re and Bi (1 g/L)) were used as internal standard and were purchased from Inorganic Ventures Inc. (Christiansburg, VA, USA) and Merck KGaA (Darmstadt, Germany). A multi-element standard solution from Agilent Technologies Inc. (Santa Clara, CA, USA) was used for instrument tuning. Argon (99.999%) as plasma gas was acquired from SOL Deutschland GmbH (Krefeld, Germany), and helium (99.996%) as collision cell gas was purchased from Linde GmbH (Pullach, Germany). Reference materials for method validation were received from DLA—Proficiency Tests GmbH (Oering, Germany) and from the Institute for Reference Materials and Measurements (Geel, Belgium). For detailed information, see Table S1 (Supporting Information).

### 2.2. Samples

In this study, 272 apple juice samples from seven different countries (Germany, DE; France, FR; Italy, IT; New Zealand, NZ; South Africa, ZA; Chile, CL; Poland, PL) and five harvest periods (2020–2024) were analyzed. Out of the 272 samples, around 85% percent of the apples were from producers, the other 15% were from private cultivation. A subset of the samples was used for method comparison between freeze-dried apple juice and fresh apple juice. Additionally, the corresponding apple marc to a subset of apple juice was analyzed. Table 1 shows an overview of the analyzed sample sizes from each country.

In a first step, the apples were washed with deionized water and then dried. Afterwards, the apples were processed into apple juice without removing the peel using a commercially available juice extractor. The juice was centrifugated, and the supernatant was frozen at −20 °C until further use. The remaining apple marc was homogenized using an ULTRA-TURRAX^®^ T18 digital (IKA-Werke GmbH & CO. KG, Staufen, Germany) and stored at −20 °C until analysis.

### 2.3. Methods

All tubes and pipette tips were pre-cleaned by soaking in 3% (*v*/*v*) nitric acid for 24 h and subsequently rinsed with ultrapure water and dried before further use.

#### 2.3.1. Lyophilization

For method comparison between freeze-dried apple juice and fresh apple juice, approximately 4 g of a subset of apple juices (see Table 1) was lyophilized. The drying process was checked daily by testing for mass constancy. The apple juice showed weight constancy after five days of freeze-drying with the equipment used in this study (Martin Christ Gefriertrocknungsanlagen GmbH, Osterode am Harz, Germany), resulting in approximately 500 mg dry matter. The lyophilized material was directly used for acid digestion. The energy consumption was measured using a commercially available electricity meter.

#### 2.3.2. Determination of Water Content

For determining the water content and calculating the dry matter of the fresh apple juice and apple marc samples, a drying scale (Smart 6, CEM, Kamp-Lintfort, Germany) was used. Approximately 700 mg of apple juice or apple marc was transferred to a fiberglass sample pad (CEM, Kamp-Lintfort, Germany) and dried until weight stability. The exact instrument parameters are given in Table S2. The analyses for each sample were carried out in triplicate. The energy consumption was measured using a commercially available electricity meter.

#### 2.3.3. Acid Digestion

For each sample approximately 5 mL apple juice, 500 mg dry matter or 1 g of apple marc was digested with 5 mL nitric acid (ROTIPURAN^®^ Supra, 69%) and 2 mL hydrogen peroxide (suprapur^®^, 30%) using the Mars 2 microwave system (CEM, Kamp-Lintfort, Germany). One commercial apple juice sample was used as quality control sample (QC sample) in each digestion batch. The program used for microwave digestion can be found in Table S3. An amount of 1 mL of a bismuth solution (0.1 mg/L in ultrapure water) was added after digestion as internal standard for evaluation purposes. An element-to-bismuth ratio was formed for all elements to assess the suitability for origin discrimination without external calibration. Finally, all samples were filled up to 12 mL with ultrapure water in pre-cleaned tubes.

#### 2.3.4. Measurements by ICP-MS

An Agilent 7800 ICP-MS from Agilent Technologies Inc. (Santa Clara, CA, USA) equipped with a quadrupole and a collision cell in combination with an ASX-500 Autosampler (Agilent Technologies Inc., Santa Clara, CA, USA) was used for the multi-elemental analyses. Fifty-three elements were included in the method: 7 Li, 9 Be, 11 B, 21 Na, 24 Mg, 27 Al, 39 K, 44 Ca, 45 Sc, 47 Ti, 51 V, 52 Cr, 55 Mn, 56 Fe, 59 Co, 60 Ni, 63 Cu, 66 Zn, 71 Ga, 75 As, 78 Se, 85 Rb, 88 Sr, 89 Y, 90 Zr, 95 Mo, 107 Ag, 111 Cd, 121 Sb, 125 Te, 137 Ba, 139 La, 140 Ce, 141 Pr, 146 Nd, 147 Sm, 151 Eu, 157 Gd, 159 Tb, 163 Dy, 165 Ho, 166 Er, 169 Tm, 172 Yb, 175 Lu, 178 Hf, 181 Ta, 193 Ir, 205 Tl, 208 Pb, 209 Bi, 232 Th and 238 U. Quantitation was performed by external calibration, corrected by internal standards (72 Ge, 103 Rh, 115 In and 185 Re) with a concentration of 1 mg/L. Measurements of 85 samples were carried out in triplicate to check which elements showed reproducible values; the rest was analyzed in single determinations. On each measurement, an instrument tuning and method tuning were carried out. Therefore, a tuning solution covering the entire mass range was used (7 Li, 59 Co, 89 Y and 205 Tl). Oxide and doubly charged ratios were checked using the same tuning solution, analyzing the 156 CeO^+^/140 Ce^+^ ratio and the 70 Ce^2+^/140 Ce^+^ ratio. Additional instrument parameters can be found in Table S4. For method validation, different reference materials were analyzed (mussel tissue (BCR^®^-668), barley grass (DLA 41/2015) and infant formula (DLA ptSU08/2022)) to check the recovery rate at different integration times and at different settings in the collision cell. The reference materials used were selected as they cover a large variation of matrices and different elements including the rare earth elements. For determination of detection and quantitation limit (LOD/LOQ), ten independent blanks were analyzed, and, afterwards, the standard deviation was calculated [27]. For collision cell optimization, the signal-to-noise ratio and the background equivalent concentration (BEC) were evaluated for every measured element in different helium gas modes (3.0–6.5 mL/min). The results are summarized in Tables S5 and S6.

### 2.4. Evaluation

The calculation of dry masses was performed with Microsoft Excel 2019 (Microsoft Corporation, Redmond, WA, USA). Agilent MassHunter 4.5 Workstation Software (version C.01.05) and Microsoft Excel 2019 were used for the calculation of element concentrations using internal and external calibration and for creation of element ratios (element-to-bismuth ratio and element-to-element ratio). Methods for variable selection (Kruskal–Wallis test and Mann–Whitney test with α = 0.15) were evaluated using the R software (version 4.1.1) and RStudio (2022.07.1). As an unsupervised multivariate data analysis method, principal component analysis (PCA) was used for the graphical presentation of sample grouping with the software Unscrambler (version 11.0, CAMO Analytics AS, Bedford, MA, USA). For origin discrimination of apple juice samples, two different supervised classification algorithms (support vector machine, SVM, and random forest, RF) were evaluated with hyperparameter tuning and method validation (repeated cross validation) using the R software (version 4.1.1) and RStudio (2022.07.1). R packages used were mlr3 (version 0.13.4), e1071 (version 1.7-11) and ranger (version 0.14.1) [28,29,30]. Different evaluation approaches regarding several origin issues at international and national level were carried out using various sample sizes. To overcome the class imbalance, the bigger classes were randomly down-sampled in relation to the minor classes of the specific research question. Table 2 gives an overview of the different evaluation approaches. For sample preparation comparison (freeze dried vs. fresh apple juice) and sample type comparison (apple juice vs. apple marc), the above-mentioned classification algorithms were used, and the accuracy of the models were compared and rated.

## 3. Results and Discussion

### 3.1. Sample Preparation Comparison (Freeze-Dried Apple Juice vs. Fresh Apple Juice)

**Figure 1:**
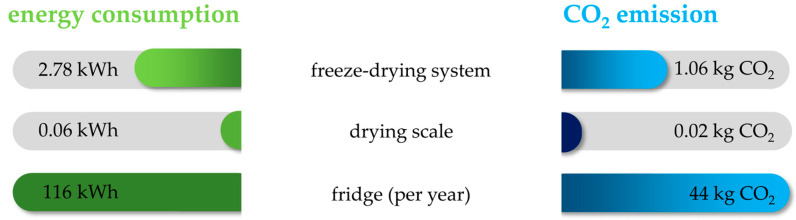
Comparison of energy consumption and CO_2_ emissions per sample when using a freeze-drying system or alternatively a drying scale. For illustrative purposes, the annual electricity consumption of an average refrigerator with a volume of 200 L is given. A measurement of 1 kWh corresponds to approx. 380 g CO_2_ emission [31,32,33].

To reduce the energy consumption and CO_2_ emission of the experiment, it was evaluated whether the use of a drying scale for calculations of element concentrations could replace the freeze-drying procedure. The freeze-drying of 5 mL apple juice causes an energy consumption of 2.78 kWh energy and is equal to 1.06 kg CO_2_ emission. Freeze-drying all 272 apple juice samples in this study would cause a consumption of 755 kWh energy and an associated CO_2_ emission of 287 kg, which is almost half of the total annual consumption of a one-person household [31]. Using a drying scale, the energy consumption and the CO_2_ emission can be reduced by a factor of around 46 compared to a freeze-drying system (Figure 1). In addition to reducing energy consumption and CO_2_ emission, the time required for sample preparation is greatly shortened by analyzing juice instead of freeze-dried material.

The dry mass of the specific sample is required to calculate the absolute element concentrations [mg/kg]. Usually, the samples used for elemental analysis are freeze-dried before digestion [15,34]. To avoid the freeze-drying process, the water content of the apple juice samples was analyzed separately using a drying scale, and the fresh apple juice was directly weighted in for digestion. To assess whether the two methods show comparable results, a subset of 85 apple juice samples from six countries (see Table 1) was analyzed using both methods. The water content determined by the two methods was compared using correlation analysis (Pearson). The water content between the two methods showed a correlation coefficient of 0.98 using an α of 0.05. The two methods thus provided very similar water contents for the samples.

**Figure 2:**
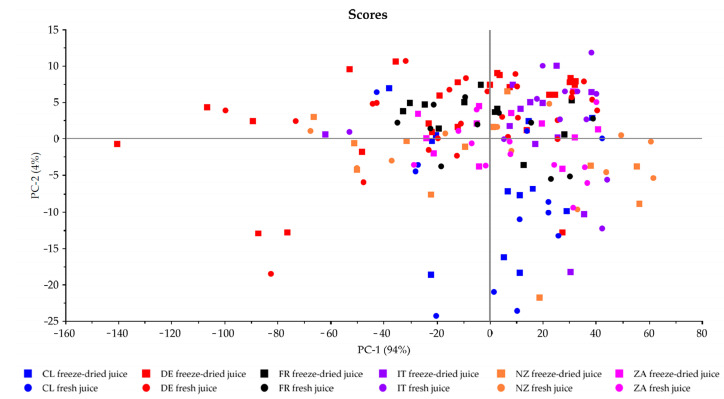
PCA for the comparison between freeze-dried apple juice samples (absolute element concentrations via freeze-drying) and fresh apple juice samples (absolute element concentrations calculated via water content).

Subsequently, the elemental profile of the freeze-dried and fresh apple juice samples was analyzed according to Section 2.3.4. To evaluate whether the results of the two methods are comparable, an unsupervised principal component analysis (PCA) was performed. Before model creation, all elements showing values below the limit of quantification (LOQ) and above the calibration range were removed (excludes Li, Be, Na, Mg, Al, K, Sc, V, Cr, Ga, As, Se, Y, Zr, Ag, Cd, Sb, Te, Ce, Pr, Nd, Sm, Eu, Gd, Tb, Dy, Ho, Er, Tm, Yb, Lu, Hf, Ta, Ir, Tl, Pb, Th and U). The PCA is shown in Figure 2 using the elements B, Ca, Ti, Mn, Fe, Co, Ni, Cu, Zn, Rb, Sr, Mo, Ba and La. All freeze-dried samples are assigned as boxes, and all fresh samples are marked as dots. The different colors represent the different countries. It becomes apparent that the element distribution between the freeze-dried and the fresh apple juice samples is very similar, and no big deviations between the two groups are visible when looking at each country individually. Using an unsupervised method such as PCA is recommendable to visualize how the samples behave without labeling them. Since no separation of the two sample preparation methods was observed, the results of the two methods are comparable.

To support this statement, a classification model with six classes (each class represents one country) was created using the element concentrations calculated with the dry masses for both sample preparation techniques (method 1). A random forest model (RF) with a repeated cross validation and without data pretreatment was used as the classification model. The model parameters can be found in Table S7. It was evaluated whether comparable classification accuracies can be achieved using both sample preparation methods. Additionally, it was checked whether the use of element ratios can help to further reduce the sample preparation effort. The use of element ratios eliminates the need for dry-mass determination and external calibration solutions. Therefore, two different element ratios were calculated. The first one (method 2) was calculated by dividing each element concentration by the corresponding bismuth concentration. Bismuth was added to each digested sample as internal standard before measurement. Bismuth can be used in this case because it is not naturally occurring in apple juice. The second element ratio approach (method 3) was constructed using the ratio of each element to each other element. Better accuracies can be achieved by using more variables for classification models [35]. In addition, different combinations of the three methods were also evaluated. The results of the different classification models are presented in Table 3. The accuracies range from 57.9% to 65.6% for the multi-class (six) model. Consequently, no major difference between the three methods is given. The best classification results for the freeze-dried juice (65.6%) and for the fresh juice (64.7%) were achieved with the absolute concentrations (method 1) in combination with the element-to-bismuth ratios (method 2). The corresponding confusion matrices can be found in Figure S1. More important than the accuracy itself is the comparability between the accuracies of the freeze-dried and the fresh juice. For the best classification model (method 1 and 2), only a 0.9 percent points (%pt) difference between the two methods occurs. The highest deviation was 4.4%pt for method 1 in combination with method 3. In summary, there is good agreement between the two sample preparation techniques. Therefore, all remaining samples were used as fresh juice, and the dry mass was determined using the drying scale, saving 508 kWh of energy and avoiding the emission of 193 kg CO_2_.

### 3.2. Origin Discrimination

In a first step, PCA models were created to exclude the influence of the crop year and the variety of the apple juice samples. No significant influence of the crop year and the variety was detected. The score plots of the corresponding PCAs are shown in Figure S2. For origin discrimination, different evaluation approaches were applied. Because of the big class imbalance between the countries, the sample size of the bigger class was randomly minimized to generate an even distribution for every model. An overview of the classification approaches and the sample sizes used for every model is shown in Table 2. For model creation, all elements above the LOQ were used as variables (B, Ca, Ti, Mn, Fe, Co, Ni, Cu, Zn, Rb, Sr, Mo, Ba, La, Ce and Nd). Variable significance was tested with the Kruskal–Wallis test for the multi-class model and with the Mann–Whitney test for two-class models (α = 0.15). The alpha level was lowered to consider more variables that are not highly significant but also not insignificant for origin discrimination. After excluding all variables with an alpha level higher than 0.15 (depending on the evaluation method), all approaches were repeated. No differences were observed regarding the results when using all variables above the LOQ. Consequently, only models using all variables above the LOQ are presented and discussed in this study. For each classification model, the data were divided into a training and test set (70/30). The process was repeated five times for each evaluation approach. Two different classification algorithms were used for every approach (random forest (RF) and support vector machine (SVM)). Moreover, a data pretreatment for the SVM model was performed using the decadic logarithm (log_10_) of the element concentrations. Analogous to Section 3.1, all three evaluation methods and their combinations were considered for each classification model. The training set was validated using a repeated cross validation. The exact method parameters for each evaluation approach are listed in Table S7. For reasons of clarity, only the best results for each evaluation approach are listed below. Selection criteria included the accuracies of the training and test sets, comparability of the two sets, standard deviation of fivefold repetition and specificity and sensitivity generated by the classification model. An overview of the results can be found in Table 4. Additional confusion matrices are shown in Figures S3 and S4.

**Figure 3:**
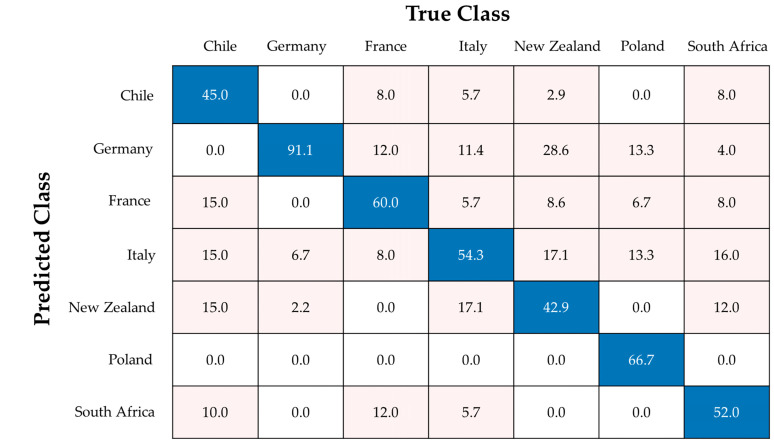
Confusion matrix showing the allocations of the test set for approach I (all countries) with a mean accuracy of 61.0%. Values are given in %. Right classifications are colored blue, wrong classifications are colored pink.

In approach I, a classification model with seven classes was created. Each class represents one country. The training set achieved an overall accuracy of 51.6% ± 4.7%, and the test set showed a mean accuracy of 61.0% ± 4.5%. The corresponding confusion matrix is shown in Figure 3. The German class showed the best sensitivity with 91.1%, and the other classes showed clearly lower sensitivities down to 42.9% for the New Zealand class. There are misclassifications between Germany and the other European samples. This leads to lower specificity values for these countries, except for the Polish class showing a specificity of 100%. The misclassifications between the European countries may be influenced by the soil on which the apple trees are grown. Due to the partly very similar soil conditions of the European countries, the change in element concentrations is less noticeable than over larger distances [36]. A similar study was performed using ^1^H NMR and apple samples from six different countries, achieving 75.8% accuracy using a RF model. Misclassifications mainly occurred also between European countries [11]. Thus, the discrimination between the European countries becomes challenging and must be further investigated (approach II and III).

**Figure 4:**
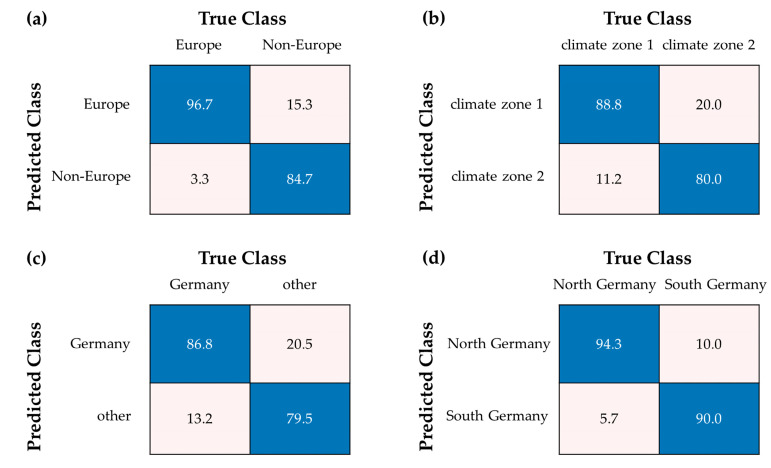
Confusion matrix of the test set showing (**a**) approach II with a mean accuracy of 90.9%; (**b**) approach III with a mean accuracy of 84.4%; (**c**) approach IV with a mean accuracy of 83.2%; (**d**) approach V with a mean accuracy of 92.3%. Values are given in %. Right classifications are colored blue, wrong classifications are colored pink.

To improve the results of the multi-class model, various two-class models were also analyzed. First, approach II was evaluated by comparing European samples with non-European samples. From the European consumer’s perspective, it is desirable to be able to distinguish between apples with shorter transportation routes and thus often lower CO_2_ emissions and apples from other origins. The test set achieved an accuracy of 90.9% ± 2.4% with a sensitivity of 96.7% for the European samples and 84.7% for the non-European samples (see Figure 4a). Therefore, the model is well suited for the detection of European samples. Other relevant non-European countries for apple growing should be added in further studies to better cover the variety of growing countries. In addition, further investigations were performed for the European market. The elemental contribution of soil in Europe is not influenced by political borders but by specific climatic conditions [36,37,38]. A separation between countries is, therefore, not always the applicable. In approach III, only European samples were analyzed, not separated by countries but by climate zones. Northern Germany and Poland formed one group (climate zone 1), whereas South Germany, France and Italy formed the second group (climate zone 2). Climate influences the distribution of elements in the soil and thus the uptake of elements in the plants. Climates were categorized based on the soil in which they grow and the weather conditions to which they are exposed and will be exposed in the future due to more extreme weather events. Temperatures in climate zone 1 are lower, whereas the temperatures in climate zone 2 are higher and will continue to rise in the future. In contrast to rising temperatures, it will probably rain less. Other studies have already identified the trend for the distribution of certain elements between these two climate zones [36,37,39]. The test set showed an accuracy of 84.4% ± 3.7% with a sensitivity of 88.8% for the samples of climate zone 1 and 80.0% for the samples of climate zone 2 (see Figure 4b). This proves the thesis that a separation along the climatic region is possible and sometimes useful. Since apples are the most frequently consumed fruit in Germany and consumers prefer to consume domestic products, the next step was to examine even smaller-scale distinctions. In approach IV, the German samples formed one group, and all foreign countries formed the second group. The test set of the classification model generated an accuracy of 83.2% ± 1.4% with a sensitivity of 86.8% for the German samples and 79.5% for the samples from the other countries (see Figure 4c). The results indicate a good suitability for the detection of German samples. As the foreign class showed a greater variance than the German class, the lower sensitivity is comprehensible. Wenck et al. [11] also used a two class-classifier to distinguish between German and foreign samples using a metabolomic approach, achieving similar results (88.5% overall accuracy with a higher sensitivity for German samples) [11]. Further, samples from two German federal states were separated into two groups in approach V. Since regionality is an increasingly important factor in the purchasing decisions of German consumers, samples from North Germany (Schleswig-Holstein) and South Germany (Baden-Wuerttemberg) were used, representing two important growing areas in Germany. The classification model achieved an accuracy of 92.3% ± 5.4% for the test set with a sensitivity of 94.3% for the North German samples and 90.0% for the South German samples (see Figure 4d). Regarding different cultivation regions, studies from other countries examined the differentiation of apples or apple juice and achieved an accuracy up to 98.6% for apple samples from two regions in China using a combination of metabolomic and elemental data [22]. Slovenian apple juice from five regions can be differentiated with an overall accuracy of 83.9% using ICP-MS in combination with IR-MS [24]. In a study from Liu et al. [25], the same techniques were used to authenticate five different regions in China, with an overall accuracy of 81.4% [25]. Compared to other studies, our local model also achieved very good results. The study by Wenck et al. [11] distinguished samples from North and South Germany using a metabolomics approach achieving an accuracy of 80.7% [11]. Consequently, the model of approach V is very well suited for the differentiation between northern and southern Germany. All classification models with the highest accuracies were generated using quantitative element concentrations in combination with a ratio-based evaluation method. Using only ratio-based data showed only slightly lower classification accuracies (1.3–4.6%) for some evaluation approaches except for approach I (see Table S8). Therefore, it is possible to minimize the experimental effort by completely omitting the dry mass and external calibration. However, to achieve the best results, ratio-based approaches must be combined with absolute element concentrations.

In summary, the presented approaches, except for approach I, provide the strong possibility to discriminate apple samples from different regions based on fresh apple juice measured with ICP-MS.

### 3.3. Sample Type Comparison (Apple Juice vs. Apple Marc)

**Figure 5:**
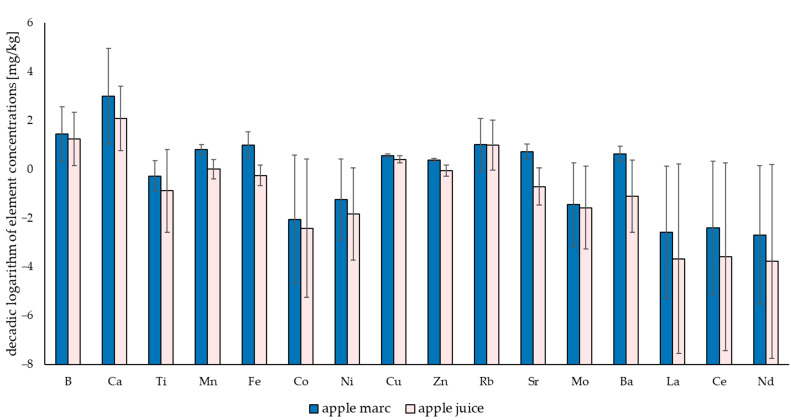
Comparison of the mean element concentrations of apple marc and apple juice samples showing the decadic logarithm of the element concentrations in mg/kg.

To verify the developed method for the origin determination of apples using apple juice, another part of the apple, the apple marc, was tested for applicability. Twelve apple marc samples (Table 1) were analyzed in triplicate to check the reproducibility. The apple marc contained nine additional elements above the LOQ (Al, V, Cd, Sb, Pr, Sm, Eu, Gd and Pb); this could be explained by the higher element concentrations in apple peel [40,41]. Three of the elements showed a high variance, and thus the reproducibility was insufficient (Sm, Gd and Pb). The comparison of the mean element concentrations of the apple juices and the corresponding apple marcs is shown in Figure 5 (only showing elements found in apple juice and apple marc). As expected, the concentrations of the apple marc samples are higher for most of the elements except for rubidium and copper, which show comparable values for both apple components. For a direct comparison, classification models were created for both apple components. Since the apple marc and the apple juice are from the same sample, comparability is given. Two-class models were used, which contained all German samples as one class (25 samples) and the foreign samples as the other class (28 samples from IT, FR and NZ). As a classification model, an SVM model with repeated cross validation and without data pretreatment was used. The model parameters can be found in Table S7. It was investigated whether comparable classification accuracies can be achieved using both sample components (apple juice and marc). The results for the classification models can be found in Table 5. The evaluation methods 1–3 were used for model creation. The accuracies of the different models showed a very good agreement between both apple components with slightly better results for the apple juice models. Additionally, the accuracies of methods 2 and 3 were as good as the results achieved with method 1, confirming the results of the previous studies (Section 3.1 and Section 3.2). A confusion matrix for an exemplary apple juice model and apple marc model can be found in Figure S5. Although the apple marc contained more elements above the LOQ, the accuracies of the classification models showed no difference between the two apple products. Consequently, the use of apple juice for origin determination is definitely recommendable.

## 4. Conclusions

In this study, 272 apple samples from seven countries were analyzed using elemental profiling with an ICP mass spectrometer. The removal of the water content, especially in the case of water-rich foods such as apples or apple juice, is usually energy- and time-consuming and costly and should be reduced to a minimum if possible. Therefore, it was first investigated whether the use of fresh apple juice for digestion and measurement could reduce the experimental effort as well as the energy consumption by eliminating the freeze-drying step. To approach this, 85 apple juice samples were processed in both ways (fresh and freeze-dried juice) and then analyzed using different evaluation methods (absolute element concentrations and ratio-based approaches).

The results showed a strong comparability between the approaches for the fresh and the freeze-dried apple juice, which is why fresh apple juice was used for the analysis in the further study. Due to the greatly reduced experimental effort, a total of 2.7 kWh of energy could be saved per sample, and the emission of 1 kg CO_2_ could be avoided. Five different authenticity issues were analyzed in which the origin discrimination of apples from different regions was examined using a support vector machine (SVM, with and without data pretreatment) and a random forest (RF) algorithm. Three different evaluation methods were used in different combinations to generate the best result for each issue. With exception of the first approach, in which each county was treated as a single class, the origin discrimination of apples using apple juice showed very good results with accuracies ranging from 83.2% to 92.3% for the test sets. It was possible to distinguish between European and non-European samples using an RF model. German samples were separated from all foreign countries using data pretreatment and an SVM model. Additionally, different climate zones can be distinguished with an accuracy of 84.4% (SVM model after data pretreatment). Since locally grown food is becoming increasingly important from a consumer’s perspective, apple samples from different regions in Germany were analyzed, showing very good accuracies (92.3%) in the SVM classification model. The best results for all issues were achieved using a combination of absolute element concentrations and a ratio-based method. Using ratio-based methods, only slightly lower accuracies (1.3–4.6%) were achieved. So, the application of ratio-based approaches is also possible, reducing the experimental effort further by eliminating the water content determination and external calibration. This study shows that elemental profiling is very well suited for distinguishing the geographical origin of apples based on juice.

In the future, the results should be confirmed by using more foreign samples, and the models should be expanded to other relevant apple-producing countries, as samples from other countries were not used to train the models. To further reduce energy consumption in this area of analysis, ratio-based evaluation approaches should be tested in further ICP-MS studies, as measurement without external calibration offers the advantage of reducing measurement time and chemical consumption. Moreover, the method should be validated with more water-rich food samples. If successful, eliminating the freeze-drying step for certain sample types will go a long way to reducing CO_2_ emissions.

## Figures and Tables

**Table 1:** Overview of the sample size used by country and evaluation approach.

Country	Sample Size Apple Juice	Sample Size Method Comparison	Sample Size Apple Marc
Germany (DE)	144	26	25
France (FR)	17	11	9
Italy (IT)	43	12	12
New Zealand (NZ)	24	12	7
South Africa (ZA)	18	12	-
Chile (CL)	15	12	-
Poland (PL)	11	-	-

**Table 2:** Overview of the different evaluation approaches, the classes and sample sizes.

Approach	Model	Classes	Sample Size
I	all countries	7	DE (30), FR (17), IT (25), NZ (24), ZA (18), CL (15), PL (11)
II	Europe vs. non-Europe	2	Europe (60), Non-Europe (57)
III	climate zones	2	North DE and PL (84), South DE, IT and FR (84)
IV	DE vs. all other countries	2	DE (128), all other countries (128)
V	North DE vs. South DE	2	North DE (25), South DE (23)

**Table 3:** Comparison of the classification accuracies of the random forest models between freeze-dried and fresh apple juice and different evaluation methods. Values and standard deviations are given in %.

Evaluation Method	Accuracy Freeze-Dried Juice [%]	Accuracy Fresh Juice [%]
1	64.6 ± 0.4	60.5 ± 1.1
2	62.5 ± 0.9	64.0 ± 0.7
3	59.6 ± 1.0	58.5 ± 1.5
1+2	65.6 ± 1.4	64.7 ± 1.1
1+3	62.3 ± 1.4	57.9 ± 0.6
2+3	59.5 ± 2.6	61.1 ± 1.2
1+2+3	62.2 ± 1.5	60.2 ± 2.4

1 = element concentrations, 2 = bismuth ratios and 3 = element ratios.

**Table 4:** Overview of the different evaluation approaches, the accuracies of training and test set and the classification algorithms used.

Evaluation Method	Accuracy Training Set [%]	Accuracy Test Set [%]	Classification Algorithm
approach I—all countries			
1+2	51.6 ± 4.7	61.0 ± 4.5	support vector machine with data pretreatment (log_10_)
approach II—Europe vs. Non-Europe			
1+2	81.1 ± 1.5	90.9 ± 2.4	random forest without data pretreatment
approach III—climate zones			
1+2+3	84.7 ± 1.6	84.4 ± 3.7	support vector machine with data pretreatment (log_10_)
approach IV—Germany vs. all other countries			
1+2	81.9 ± 1.4	83.2 ± 1.4	support vector machine with data pretreatment (log_10_)
approach V—North vs. South Germany			
1+2	86.0 ± 3.8	92.3 ± 5.4	support vector machine without data pretreatment

1 = element concentrations, 2 = bismuth ratios and 3 = element ratios.

**Table 5:** Comparison of the classification accuracies of the support vector machine models between apple juice and apple marc and different evaluation methods. Values and standard deviation are given in %.

Method	Accuracy Apple Juice [%]	Accuracy Apple Marc [%]
1	83.0 ± 1.1	83.1 ± 2.0
2	87.9 ± 1.5	83.7 ± 0.9
3	85.8 ± 1.7	84.6 ± 2.0

## Data Availability

The original contributions presented in the study are included in the article/Supplementary Material, further inquiries can be directed to the corresponding author.

## Supplementary Materials

The following supporting information can be downloaded at https://www.mdpi.com/article/10.3390/foods13233783/s1: Table S1. Overview of chemicals and solutions used in this study; Table S2. Time and temperature progress during sample drying of the apple juice and apple marc samples using the Smart 6 drying scale; Table S3. Time and temperature progress during sample digestion of the apple juice and apple marc samples using Mars 2; Table S4. Instrument conditions and measurement parameters for the Agilent 7800 used in this study; Table S5. Summary of the measured isotopes, the helium modus and integration time used for analysis and the limit of detection (LOD) and quantification (LOQ) for each element; Table S6. Overview of the measured reference materials’ infant formula (DLA ptSU08/2022), barley grass (DLA 41/2015) and mussel tissue (BCR^®^-668), showing the recovery rates for the specific elements in %; Table S7. Overview of the different evaluation approaches, classification algorithm, model parameters and validation parameters used in this study; Table S8. Overview of the classification accuracies for the different evaluation methods of the best evaluation approach. Values given in %; Figure S1. (a) Confusion matrix for RF classification model without data pre-treatment, with an accuracy of 65.6% using method 1 and 2 and freeze-dried apple juice. (b) Confusion matrix for RF classification model without data pre-treatment, with an accuracy of 64.7% using method 1 and 2 and fresh apple juice. Values are given in %; Figure S2. (a) PCA showing the element concentrations of apple juice samples after centering and scaling, colored by variety. Only varieties with four or more apple juice samples are shown. (b) PCA showing the element concentrations of apple juice samples colored by crop year. Crop year 2024 is not shown because of the small sample amount from this year; Figure S3. Confusion matrix showing the allocations of the training set for approach I (all countries) with a mean accuracy of 51.6%. Values are given in %; Figure S4. Confusion matrix of the training set showing (a) approach II with a mean accuracy of 81.1%; (b) approach III with a mean accuracy of 84.7%; (c) approach IV with a mean accuracy of 81.9%; (d) approach V with a mean accuracy of 86.0%. Values are given in %; Figure S5. (a) Confusion matrix for SVM classification model without data pre-treatment, with an accuracy of 87.9% using method 2 and apple juice. (b) Confusion matrix for SVM classification model without data pre-treatment, with an accuracy of 83.7% using method 2 and apple marc. Values are given in %.
