# Supplementary material for: Food Authentication Goes Green: Method Optimization for Origin Discrimination of Apples Using Apple Juice and ICP-MS
Source: Foods. 2024 Nov 25;13(23):3783. doi: 10.3390/foods13233783 (PMC11639750; doi:10.3390/foods13233783)
Supplement: Supplementary file 1 [file foods-13-03783-s001.zip › foods-3292115-supplementary.pdf]

Supporting Information

Table S1. Overview of chemicals and solutions used in this study.

reagent	comment	manufacturer
water, ultrapure	> 18 MΩ	Direct-Q purifying system, Merck Millipore Inc., (Billerica, MA, USA)
nitric acid (HNO_3)	ROTIPURAN Supra, 69%, *v/v*	Carl Roth GmbH & Co. KG (Karlsruhe, Germany)
hydrogen peroxide (H_2O_2)	suprapur, 30%, v/v	Merck KGaA (Darmstadt, Germany)
standard solutions	- 10 mg/L: Li, Na, Mg, Al, K, V, Cr, Mn, Co, Ni, Cu, Ga, Rb, Sr, Mo, Ag, Cd, Te, Ba, Tl, Pb, Bi and U (used in a range from 0.001 - 2000 µg/L)<br>- 100 mg/L: Be, B, Fe, Zn, As, and Se (used in a range from 0.01 - 20.000 µg/L)<br>- 1,000 mg/L: Ca (used in a range from 0.1 - 200.000 µg/L)	Merck KGaA (Darmstadt, Germany)
	1 g/L: Ge, Rh, In, and Re (used in 100 µg/L)	Inorganic Ventures Inc. (Christiansburg, VA, USA)
	100 mg/L: Hf, Ir, Sb, Sn, Ta, Ti, Zr (used in a range from 0.001-100 µg/L)	Carl Roth GmbH & Co. KG (Karlsruhe, Germany)
	10 mg/L: Sc, Y, La, Ce, PR, Nd, Sm, Eu, Gd, Tb, Dy, Ho, Er, Tm, Yb, Lu, Th (used in a range from 0.001-10 µg/L)	PerkinElmer Inc. (Waltham, MA, USA)
	1 g/L: Bi (used in 0.1 mg/L)	Merck KGaA (Darmstadt, Germany)
tuning solution	10 µg/L: Li, Y, Tl, Ce, Co (used in 1µg/L)	Agilent Technologies Inc. (Santa Clara, CA, USA)
reference material	DLA 41/2015 (barely grass), DLA ptSU08/2022 (infant formula)	DLA-Proficiency Tests GmbH (Oering, Germany)
	BCR®-668 (mussel tissue)	Institute for Reference Materials and Measurements (Geel, Belgium)
argon	≥99.999%	SOL Deutschland GmbH (Krefeld, Germany)
helium	≥99.996%	Linde GmbH (Pullach, Germany)

Table S2. Time and temperature progress during sample drying of the apple juice and apple marc samples using the Smart 6 drying scale.

level	time [min]	temperature [°C]	magnetic power [W]	infrared power [W]
1	0.45	105	100	50
2	max. 10	105	50	100

Table S3. Time and temperature progress during sample digestion of the apple juice and apple marc samples using the Mars 2.

time [min]	temperature [°C]
0	20
25	190
40	190
55	20

Table S4. Instrument conditions and measurement parameters for the Agilent 7800 used in this study.

parameter	ICP-MS
**forward power (W)**	1500
**plasma gas flow rate (L/min)**	15.0
**auxiliary gas flow rate (L/min)**	0.90
**nebulizer gas flow rate (L/min)**	0.75
**sample uptake (µL/min)**	200
**cones**	Ni
**nebulizer**	MicroMistTM
**number of acquisition replica**	3
**tuning**	masses: 7/89/205<br>doubly charged ratio: Ce^{++}/Ce^{+} (70/140); oxide ratio: CeO^{+}/Ce^{+} (156/140)
**selected isotopes for internal standard**	72Ge, 103Rh, 115In, 185Re
**correction equations**	115In=115In-0.0149·Sn118; Pb=206Pb+207Pb+208Pb

Table S5. Summary of the measured isotopes, the helium modus and integration time used for analysis and the limit of detection (LOD) and quantification (LOQ) for each element.

element	*m/z*	helium modus [mL/min]	Integration time [s]	LOD [µg/L]	LOQ [µg/L]
Li	7	0.0	0.6999	0.0793	0.2403
Be	9	0.0	0.0999	0.0181	0.0549
B	11	3.0	0.0999	5.8733	17.7978
Na	23	3.5	0.0999	0.3780	1.1455
Mg	24	3.0	0.0999	0.1852	0.5611
Al	27	0.0	0.6999	0.6237	1.8900
K	39	3.0	0.0999	2.6420	8.0060
Ca	44	4.0	0.0999	23.1743	70.2253
Sc	45	4.5	0.0999	0.0171	0.0519
Ti	47	3.0	0.0999	0.0428	0.1297
V	51	4.5	0.5001	0.0037	0.0111
Cr	52	4.0	0.0999	0.0139	0.0420
Mn	55	4.0	0.0999	0.0084	0.0256
Fe	56	5.5	0.0999	0.0817	0.2477
Co	59	5.0	0.3000	0.0026	0.0080
Ni	60	3.5	0.3000	0.0070	0.0212
Cu	63	4.5	0.0999	0.0410	0.1243
Zn	66	4.5	0.0999	0.9840	2.9819

element	*m*/*z*	helium modus [mL/min]	Integration time [s]	LOD [µg/L]	LOQ [µg/L]
Ga	71	4.0	0.0999	0.0041	0.0125
As	75	4.0	0.3000	0.6055	1.8349
Se	78	5.0	0.0999	0.9448	2.8632
Rb	85	4.5	0.0999	0.0177	0.0536
Sr	88	5.5	0.0999	0.1027	0.3112
Y	89	5.5	0.0999	0.0018	0.0054
Zr	90	3.5	0.0999	0.0139	0.0422
Mo	95	4.0	0.3000	0.1572	0.4763
Ag	107	3.0	0.0999	0.0447	0.1354
Cd	111	5.5	0.0999	0.0160	0.0485
Sb	121	4.0	0.0999	0.0080	0.0243
Te	125	3.0	0.0999	0.0201	0.0610
Ba	137	5.5	0.0999	0.0287	0.0871
La	139	0.0	0.5001	0.0003	0.0008
Ce	140	0.0	0.5001	0.0005	0.0016
Pr	141	0.0	0.5001	0.0001	0.0004
Nd	146	0.0	0.5001	0.0007	0.0020
Sm	147	0.0	0.5001	0.0007	0.0020
Eu	151	0.0	0.5001	0.0002	0.0006
Gd	157	0.0	0.5001	0.0008	0.0023
Tb	159	0.0	0.6999	0.0001	0.0004
Dy	163	0.0	0.5001	0.0005	0.0015
Ho	165	0.0	0.6999	0.0001	0.0003
Er	166	0.0	0.5001	0.0005	0.0014
Tm	169	0.0	0.5001	0.0002	0.0005
Yb	172	0.0	0.5001	0.0004	0.0012
Lu	175	0.0	0.5001	0.0001	0.0004
Hf	178	0.0	0.0999	0.0018	0.0054
Ta	181	0.0	0.3000	0.0036	0.0108
Ir	193	0.0	0.0999	0.0114	0.0345
Tl	205	0.0	0.0999	0.4460	1.3515
Pb	208	0.0	0.6999	0.0090	0.0274
Bi	209	0.0	0.0999	0.0189	0.0571
Th	232	0.0	0.5001	0.0003	0.0008
U	238	0.0	0.6999	0.0028	0.0085

Table S6. Overview of the measured reference materials infant formula (DLA ptSU08/2022), barley grass (DLA 41/2015) and mussel tissue (BCR®-668), showing the recovery rates for the specific elements in %.

element	recovery rate [%] infant formula	recovery rate [%] barley grass	recovery rate [%] mussel tissue
Na	98.0	92.0	-
Mg	97.7	102	-
Al	69.9	78.2	-
K	100	100	-
Ca	99.3	100	-
Sc	-	-	105
Cr	107	101	91.6
Mn	98.8	101	-
Fe	101	102	-
Ni	99.2	82.1	-
Cu	99.5	101	-
Zn	91.5	100	100
As	103	-	116
Se	99.5	-	-
Rb	-	106	-
Y	-	-	100
Mo	-	105	114
Cd	96.7	99.5	104
Ba	107	101	-
La	-	88.3	107
Ce	-	98.4	109
Pr	-	-	109
Nd	-	87.0	108
Sm	-	-	111
Eu	-	-	101
Gd	-	-	110
Tb	-	-	103
Dy	-	-	98.8
Ho	-	-	96.0
Er	-	-	100
Tm	-	-	96.1
Yb	-	-	97.4
Lu	-	-	98.6
Pb	95.3	88.7	-
Th	-	-	101
U	-	-	104

Table S7. Overview of the different evaluation approaches, classification algorithm, model parameters and validation parameters used in this study.

approach	classification algorithm	model paramters	validation
sample preparation comparison	random forest	importance = permutation, impurity, impurity correction, none; trees = 500 - 5000; mtry = variable	repeated cross validation, folds = 4, repeats = 5
origin approach I	support vector machine	radial kernel, cost = 80, gamma = 0.05	repeated cross validation, folds = 4, repeats = 5
origin approach II	random forest	importance = impurity, trees = 500, mtry = 1	repeated cross validation, folds = 5, repeats = 5
origin approach III	support vector machine	radial kernel, cost = 30, gamma = 0.01	repeated cross validation, folds = 5, repeats = 5
origin approach IV	support vector machine	radial kernel, cost = 20, gamma = 0.06	repeated cross validation, folds = 5, repeats = 5
origin approach V	support vector machine	radial kernel, cost = 40, gamma = 0.06	repeated cross validation, folds = 4, repeats = 5
sample type comparison	support vector machine	Polynomial or radial kernel, cost = variable, gamma = variable	repeated cross validation, folds = 5, repeats = 5

Table S8. Overview of the classification accuracies for the different evaluation methods of the best evaluation approach. Values given in %.

Evaluation approach	method 1 [%]	method 2 [%]	method 3 [%]	method 1+2 [%]	method 1+3 [%]	method 2+3 [%]	method 1+2+3 [%]
			**training set**				
I	49.8	44.3	44.5	51.6	46.4	46.1	47.2
II	80.9	76.1	83.7	81.1	84.2	84.0	84.3
III	78.3	76.9	80.3	82.9	82.6	81.9	84.7
IV	75.9	75.4	78.6	81.9	79.9	81.2	81.4
V	80.2	83.2	71.2	85.9	75.2	74.7	75.4
			**test set**				
I	56.5	49.0	47.0	61.0	50.5	50.0	53.0
II	88.0	86.3	83.4	90.9	84.0	83.4	85.1
III	76.0	78.4	77.2	88.0	80.0	82.4	84.4
IV	74.5	78.7	79.7	83.2	81.8	81.8	82.6
V	83.1	92.3	70.8	92.3	75.4	72.3	78.5

(a)

True Class (columns) / **Predicted Class** (rows)

	Chile	Germany	France	Italy	New Zealand	South Africa
Chile	75.0	0.6	9.1	3.3	0.0	6.7
Germany	8.3	74.9	42.5	21.0	37.0	3.3
France	3.3	7.2	38.5	4.0	0.3	9.0
Italy	0.0	8.2	0.4	63.3	16.7	0.0
New Zealand	0.0	7.7	0.0	1.3	46.0	0.0
South Africa	13.3	1.4	9.5	7.0	0.0	81.0

(b)

True Class (columns) / **Predicted Class** (rows)

	Chile	Germany	France	Italy	New Zealand	South Africa
Chile	63.7	5.1	5.1	4.3	0.0	5.3
Germany	10.7	80.3	43.6	12.7	34.0	11.0
France	3.3	8.3	30.5	0.0	1.0	11.3
Italy	0.0	2.0	6.2	77.7	8.3	2.7
New Zealand	6.0	2.3	0.4	4.3	46.3	0.3
South Africa	16.3	2.0	14.2	1.0	10.3	69.3

Figure S1. (a) Confusion matrix for RF classification model without data pre-treatment, with an accuracy of 65.6% using method 1 and 2 and freeze-dried apple juice. (b) Confusion matrix for RF classification model without data pre-treatment, with an accuracy of 64.7% using method 1 and 2 and fresh apple juice. Values are given in %.

(a)

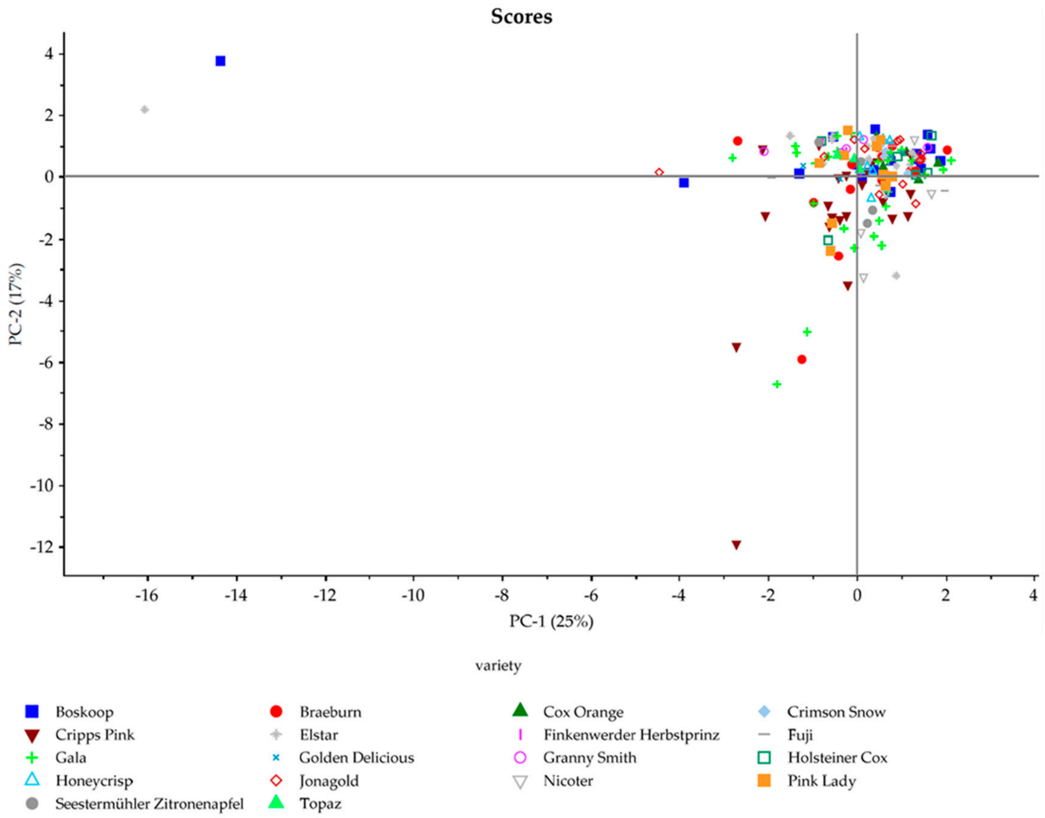

(b)

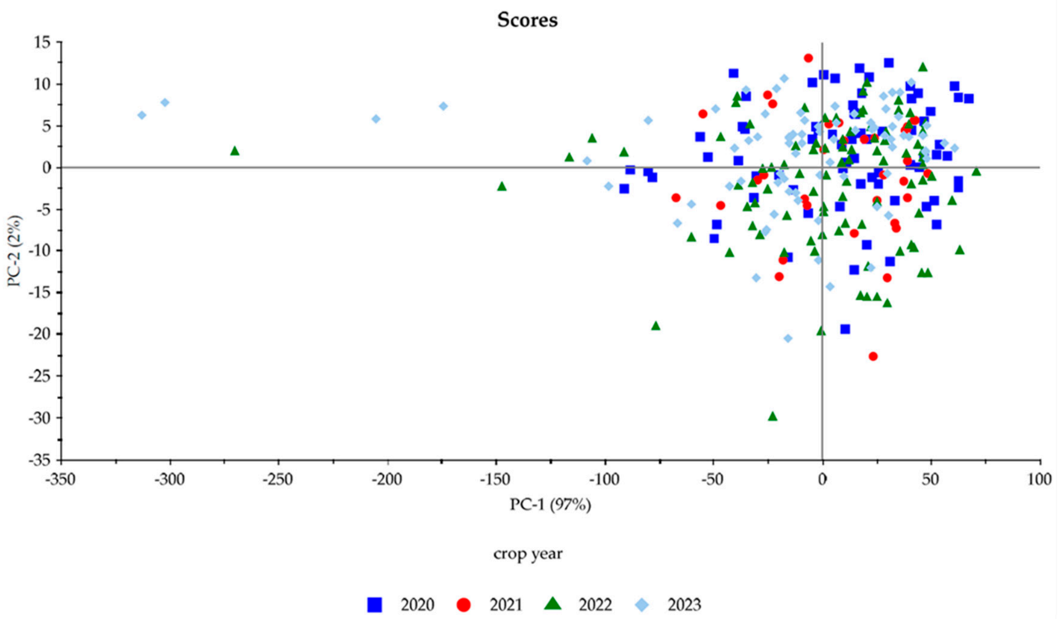

Figure S2. (a) PCA showing the element concentrations of apple juice samples after centering and scaling, colored by variety. Only varieties with four or more apple juice samples are shown. (b) PCA showing the element concentrations of apple juice samples colored by crop year. Crop year 2024 is not shown because of the small sample amount from this year.

True Class (columns) / **Predicted Class** (rows)

Predicted \ True	Chile	Germany	France	Italy	New Zealand	Poland	South Africa
Chile	42.9	0.0	16.7	5.8	4.0	0.0	7.1
Germany	2.9	90.9	2.7	23.1	14.6	40.5	8.0
France	21.5	0.0	36.0	7.1	3.8	6.0	12.6
Italy	9.5	3.8	14.3	42.4	23.1	9.5	11.4
New Zealand	10.9	3.4	6.0	16.9	45.9	13.5	11.7
Poland	0.0	0.6	2.3	0.0	1.2	20.5	0.0
South Africa	12.4	1.3	22.0	4.7	7.5	10.0	49.2

Figure S3. Confusion matrix showing the allocations of the training set for approach I (all countries) with a mean accuracy of 51.6%. Values are given in %.

(a) True Class / Predicted Class

Predicted \ True	Europe	Non-Europe
Europe	85.9	24.0
Non-Europe	14.1	76.0

(b) True Class / Predicted Class

Predicted \ True	climate zone 1	climate zone 2
climate zone 1	89.5	20.0
climate zone 2	10.5	80.0

(c) True Class / Predicted Class

Predicted \ True	Germany	other
Germany	85.4	21.6
other	14.6	78.4

(d) True Class / Predicted Class

Predicted \ True	North Germany	South Germany
North Germany	86.0	14.1
South Germany	14.0	85.9

Figure S4. Confusion matrix of the training set showing (a) approach II with a mean accuracy of 81.1%. (b) approach III with a mean accuracy of 84.7%. (c) approach IV with a mean accuracy of 81.9%. (d) approach V with a mean accuracy of 86.0%. Values are given in %.

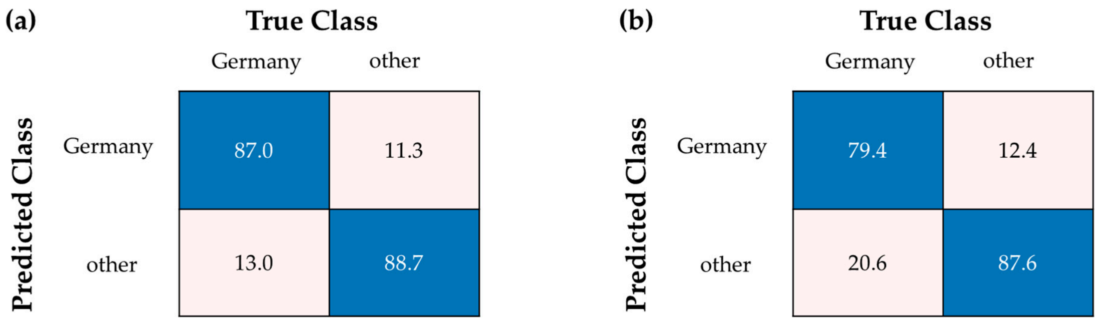

Figure S5. (a) Confusion matrix for SVM classification model without data pre-treatment, with an accuracy of 87.9% using method 2 and apple juice. (b) Confusion matrix for SVM classification model without data pre-treatment, with an accuracy of 83.7% using method 2 and apple marc. Values are given in %.
